# Perceived psychological stress and associated factors in the early stages of the coronavirus disease 2019 (COVID-19) epidemic: Evidence from the general Chinese population

**DOI:** 10.1371/journal.pone.0243605

**Published:** 2020-12-04

**Authors:** Xiao Yang, Zhenzhen Xiong, Zhixiong Li, Xiao Li, Weiyi Xiang, Yiwen Yuan, Zhe Li

**Affiliations:** 1 Mental Health Center and Psychiatric Laboratory, The State Key Laboratory of Biotherapy, West China Hospital, Sichuan University, Chengdu, Sichuan, China; 2 West China Brain Research Center, West China Hospital, Sichuan University, Chengdu, Sichuan, China; 3 School of Nursing, Chengdu Medical College, Chengdu, Sichuan, China; 4 The Third Department of Clinical Psychology, Karamay Municipal People's Hospital, Karamay, Xinjiang, China; 5 The West China College of Medicine, Sichuan University, Chengdu, Sichuan, China; 6 The Mental Health Center and National Clinical Research Center for Geriatrics, West China Hospital, Sichuan University, Chengdu, Sichuan, China; Qazvin University of Medical Sciences, ISLAMIC REPUBLIC OF IRAN

## Abstract

**Introduction:**

Coronavirus disease 2019 (COVID-19) is an acute respiratory infection caused by novel coronavirus 2019. Many individuals suffered psychological symptoms in the early stage when the epidemic was uncertain. We explored the perceived psychological stress and associated factors in the early stage of COVID-19 epidemic.

**Method:**

The Perceived Stress Scale, Simplified Coping Style Questionnaire, Social Support Rating Scale and a general information questionnaire were integrated in an on-line survey conducted from February 1, 2020 until February 4, 2020. Multiple linear regression analysis was performed to explore whether coping style, social support or other factors contributed to psychological stress.

**Results:**

A total of 1638 participants were included, of whom 44.3% showed moderate psychological stress. Individuals who were younger, female, unmarried, spent more time on the disease, felt more concern about it, reported lower social support (Subjective Social support; Objective social support; Utilization social support), or showed a negative coping style were more likely to suffer higher psychological stress in the early stages of the COVID-19 epidemic.

**Conclusion:**

Psychological interventions may be targeted to individuals with the risk characteristics identified in this study. It may be helpful to promote social support and positive coping style in the early stage of infectious disease epidemics. This initial evidence from the general Chinese population may be relevant to interventions in other countries for dealing with the COVID-19 and other epidemics.

## Introduction

In December 2019, several patients manifested an unknown pneumonia in Wuhan, China [1]. In January 2020, the disease was confirmed to be caused by a novel virus that could be transmitted between people and for which only symptomatic treatments and no vaccine were available. Safety measures such as school closures, transport bans and workplace shutdowns helped to limit spread of COVID-19 in Wuhan, and similar measures were soon launched in other cities [2]. Afterwards, it became a public health emergency of international concern, as the World Health Organization declared.

Previous studies found that in comparable epidemics of infectious diseases such as the severe acute respiratory syndrome (SARS), people may suffer psychological problems and may benefit from psychological interventions [3, 4]. In the early stage of the COVID-19 epidemic, when little was known about the virus and the disease, the general population may have suffered psychological stress about becoming infected or spreading the virus to their families, friends, or colleagues [5]. Psychological well-being can be affected by many factors [6] such as coping styles, which can determine risk of psychological problems or mental illnesses, including post-traumatic stress disorder (PTSD), anxiety, and depression [7, 8]. Effective and sufficient social support can show positive effects on sleep quality [9] and mental health [10, 11] during epidemics of infectious diseases. During the SARS epidemic, social support, including different types of verbal and behavioral responses, helped preserve psychological status, especially during the acute stage [3]. Similarly, research during the present COVID-19 epidemic has found that social and emotional support as well as shared empathy from friends or family members can help reduce anxiety and stress, and improve self-efficacy [12].

To counteract psychological stress during epidemics of infectious disease, psychological intervention [13] and timely mental health care [2] can be effective. In this study, we investigated the perceived psychological stress and explored whether coping style, social support or other factors contributed to psychological stress in the Chinese general population during the early stages of the COVID-19 epidemic. The results are aimed at providing insights to guide mental health care and targeted psychological interventions in the early stages of this and similar epidemics.

## Participants and methods

### Participants

All invitees completed the online survey anonymously via Questionnaire Star (www.wjx.cn) from 4 p.m. (Beijing time) on February 1, 2020 until midnight on February 4, 2020. Snowball sampling was used to invite potential study participants. Through the WeChat application, which constitutes a mainstream media in China, the investigators invited an initial group of 10 individuals with different age, education level etc. to participate. The first set of invitees then forwarded the invitations to 10 companions whom they considered suitable. The second set were then asked to forward the survey in the same way. The study included a general population aged 18 years or older who volunteered to participate in the study. All participants received a complete description of this survey and were asked to sign an online informed consent prior to data collection. Respondents were excluded if they had been diagnosed with any DSM-IV (Diagnostic and Statistical Manual of Mental Disorders, 4th edition) disorder before this survey. This study was approved by the Ethics Committee of West China Hospital, Sichuan University (No. 2020–178).

### Questionnaires

Using a custom-designed survey, we collected general and epidemic information that included: age, sex, marital status, education level, history of visiting epidemic areas recently, the presence of infected cases in the respondent’s community, how many hours he or she spent thinking about COVID-19 every day, and his or her concern about COVID-19. Psychological stress, social support and coping style were assessed using the Perceived Stress Scale (PSS-10) [14], Social Support Rate Scale (SSRS) [15] and the Simplified Coping Style Questionnaire (SCSQ) [16].

The PSS is a popular self-report instrument developed to assess the perceived stress of participants during the previous month [14]. The 10-item version (PSS-10) exhibits good reliability and is widely used to measure “how unpredictable, uncontrollable, and overloaded respondents find their lives”. Respondents answer each item of the questionnaire using a Likert-type scale from 0 (never) to 4 (very often). Total scores range from 0 to 40, and 0–14 means mild psychological stress; 15–25, moderate stress; 26–31, severe stress; and 32–40, very severe stress. The Cronbach’s α for internal consistency has been reported as 0.78 [17].

The SSRS is a 10-item self-report instrument to measure the type and level of social support received by individuals [15]. It consists of 10 questions and three subscales including objective support (3 items), subjective support (4 items) and support utilization (3 items). Objective support means practical, tangible and direct support or resources that one receives. Subjective support means perceived support such as the feeling that one has been helped, cared for and supported by others. Utilization of support refers to the degree of support perceived. A higher score on each subscale corresponds to greater social support. Cronbach’s α for internal consistency of the SSRS has been reported to be 0.896 [15].

The SCSQ is a 20-item self-report instrument to assess an individual’s coping style. It consists of two subscales, positive coping style (12 items) and negative coping style (8 items) [16]. Respondents answer each item on the questionnaire using a Likert scale (0, never; 1, seldom; 2, often; 3, always). Scores are calculated for each subscale, and the tendency toward positive or negative coping style is determined using an equation [18]: respondents whose tendency is greater than 0 tend to adopt a positive coping style when faced with stress, while those with a tendency less than 0 tend to adopt a negative coping style. The Cronbach’s α for internal consistency of the SCSQ has been reported to be 0.82 [16].

### Quality control

The same Internet Protocol address was permitted to be used only once to finish the survey. Surveys on which respondents spent fewer than 120 seconds were regarded as invalid and excluded. No personal or identifying information were collected on this survey.

### Statistical analysis

Statistical analyses were performed using SPSS 24.0 for Macintosh (IBM, Chicago, IL, USA). Outliers were checked and removed if they had a value more than 3 standard deviation away from mean value. The continuous data was described by mean value and categorical data was described by constituent ratio or frequency. Multiple linear regression analysis was conducted to identify variables predictive of, or associated with, psychological stress in the early stages of COVID-19 epidemic. The multiple linear regression analysis was constructed in a stepwise fashion with the following covariates: age, gender, education level, marital status, history of visiting the epidemic areas recently, infected case in community, history of visiting an epidemic area, presence of infected cases in the respondent’s community, time spent thinking about COVID-19 per day, level of concern about COVID-19, coping style, and questionnaire scores on subjective support, objective support and utilization of support. Differences associated with *p* < 0.05 were considered to be statistically significant.

## Results

### Demographic information

A total of 1642 participants responded to the online survey, and 1638 individuals were included in the final analysis. Their average age was 33.84±12.28 years, 66.91% were women, and 43.04% were unmarried. The average time respondents spent thinking about the disease was 3.92±3.52 hour every day. The average score on the PSS was 14.33±6.71, and 51.04% of respondents showed mild psychological stress, 44.32% moderate stress and 4.46% severe stress. Most respondents (76.80%) reported being extremely concerned about the disease, and 33.64% showed a negative coping style (Table 1).

**Table 1:** Demographic information of the sample (n = 1638).

Variables	Mean / N (%)
**Age**	33.84±12.28
**Gender**	
male	542 (33.09)
female	1096 (66.91)
**Marital status**	
unmarried	705 (43.04)
married	933 (56.96)
**Education level**	
primary school	5 (0.31)
middle school	45 (2.75)
high school	90 (5.50)
technical qualification	357 (21.80)
bachelor’ degree	928 (56.66)
graduate	213 (13.00)
**History of visiting the epidemic areas recently**	
no	1314 (80.22)
yes	324 (19.78)
**Infected case in community**	
no	1297 (79.18)
yes	341 (20.82)
**Time spending about COVID-19 everyday (hours)**	3.92±3.52
**Concern about COVID-19**	
not concerned	1(0.10)
less concerned	29 (1.77)
concerned	68 (4.15)
more concerned	282 (17.22)
extremely concerned	1258 (76.80)
**Score of perceived stress scale**	14.33±6.71
0–14	836 (51.04)
15–25	726 (44.32)
26–31	73 (4.46)
32–40	3 (0.18)
**Subjective social support**	19.43±6.69
**Objective social support**	7.92±3.92
**Utilization social support**	7.08±2.41
**Coping style**	
negative	551 (33.64)
positive	1087 (66.36)

### Multiple linear regression analysis

All factors’ values in the multiple linear regression analysis were listed in Table 2. The multiple linear regression analysis explained 50.9% of the variation in psychological stress (R = 0.715, adjusted R^2^ = 0.509). The multiple linear regression analysis showed that, after adjustment, individuals who were younger (*B* = -0.083, *p*<0.001, 95%CI: (-0.108~ -0.057)), female (*B* = 0.973, *p*<0.001, 95%CI: (0.481~ 1.466)), unmarried (*B* = 1.221, *p*<0.001, 95%CI: (0.576~1.865)) spent more time on the disease (*B* = 0.203, *p*<0.001, 95%CI: (0.130~0.277)), felt more concern about it (*B* = 0.643, *p*<0.05, 95%CI: (0.281~1.005)), reported lower social support (Subjective Social support (*B* = -0.236, *p*<0.001, 95%CI: (-0.298~ -0.174)); Objective social support (*B* = -0.205, *p*<0.001, 95%CI: (-0.298~ -0.111)); Utilization social support (*B* = -0.270, *p*<0.001, 95%CI: (-0.411~ -0.128)), or showed a negative coping style (*B* = -3.934, *p*<0.001, 95%CI: (-4.575~ -3.293)) were more likely to suffer higher psychological stress in the early stages of the COVID-19 epidemic (Table 3).

**Table 2:** Factors’ values assigned in multiple linear regression analysis (n = 1638).

Variables	Value
**Age**	Original continuous value
**Gender**	0 = Male, 1 = Female
**Education level**	0 = primary school, 1 = middle school, 2 = high school, 3 = technical qualification, 4 = bachelor’ degree, 5 = graduate
**Marital status**	0 = Married, 1 = Unmarried
**History of visiting the epidemic areas recently**	0 = No, 1 = Yes
**Time spending about COVID-19 everyday**	Original continuous value
**Concern about COVID-19**	Original continuous value
**Infected case in community**	0 = No, 1 = Yes
**Subjective social support**	Original continuous value
**Objective social support**	Original continuous value
**Utilization social support**	Original continuous value
**Coping style**	0 = Negative, 1 = Positive

**Table 3:** Multiple linear regression analysis for psychological stress for perceived psychological stress (n = 1638).

Dependent Variables	Independent variable	Unstandardized B score	Partial eta squared	t	p	95%CI
**Perceived psychological stress (R = 0.715, Adjusted R**^**2**^ **= 0.509)**	Age	-0.083	0.024	-6.377	<0.001	-0.108~ -0.057
	Gender	0.973	0.009	3.876	<0.001	0.481~1.466
	Marital status	1.221	0.008	3.714	<0.001	0.576~1.865
	Time spending about COVID-19 everyday	0.203	0.018	5.414	<0.001	0.130~0.277
	Concern about COVID-19	0.643	0.007	3.487	<0.05	0.281~1.005
	Subjective social support	-0.236	0.033	-7.470	<0.001	-0.298~ -0.174
	Objective social support	-0.205	0.011	-4.299	<0.001	-0.298~ -0.111
	Utilization social support	-0.270	0.008	-3.726	<0.001	-0.411~ -0.128
	Coping style	-3.934	0.082	-12.038	<0.001	-4.575~ -3.293

## Discussion

In this study, we found that about half of respondents from the general Chinese population showed moderate psychological stress, and about 4.46% showed severe psychological stress. We also found that individuals who were younger or unmarried, spent more time on the disease, felt greater concern over it, reported lower social support, or showed negative coping style were more likely to suffer higher psychological stress in the early stages of the COVID-19 epidemic. Psychological interventions targeted to such individuals may help preserve or improve their psychological status in the early stages of a similar outbreak of infectious disease.

The prevalence of higher psychological stress in this study suggests that the outbreak has placed a mental health burden on the general population in China. Perceived psychological stress and stress-related factors may increase risk of mental conditions such as depression, anxiety and PTSD [19, 20]. Our middle-aged respondents reported higher stress, consistent with reports that middle-aged individuals are more likely than elderly to perceive problems as trouble and challenges [21]. People in middle age are usually exposed to diverse stressors including caring for children and elderly parents, financial pressure, and problems at work and personal relationships [22, 23]. These may further contribute to their perceived stress during the outbreak. In this study, females were found to be more likely to suffer higher psychological stress. This result was consistent with the previous study, which also found that female may be associated with the worse psychological status during COVID-19 epidemic [24, 25]. Women may be more likely to have sleep problem, depressive symptom [26], and more intrusive flashbacks as they were more sensitivity to emotional stimuli and resulted in altered immune function and hormone level [25, 27]. We also found that unmarried respondents were more likely to suffer higher pressure. Analogously, a previous study reported that lower family cohesion and marriage quality were associated with higher anxiety and depression [28].

We also found that individuals who spent more time occupied with COVID-19 or who were more concerned about the disease reported higher perceived stress. Recent research found that 88.97% of Chinese adults use WeChat to obtain information about the COVID-19 outbreak [29]. During the early stage of the epidemic, the causative virus was repeatedly described on WeChat as a “killer virus”, propagating a sense of danger and uncertainty among the public [2]. In the early stage of the epidemic, human-to-human transmission was established, no vaccine was available, several cities were put on lockdown, and the epidemic was declared to be an international public health emergency [30]. In addition, many differences were reported between COVID-19 and SARS in clinical characteristics, fatality rate and other epidemiological characteristics, while the causative viruses were reported to show different transmission routes and incubation periods [31]. Thus, individuals who spent more time occupied with COVID-19 in the early stage of the epidemic were likely exposed to a substantial amount of negative or panic-inducing information about the epidemic, which may have contributed to their psychological stress.

Social support is defined as an individual’s belief that he or she is cared about, loved, and valued and that assistance is available to him or her, regardless of whether support is actually available [32]. Social support is considered to help protect individuals from stress [19]. Poor social support has been associated with many mental health problems such as depression [33], anxiety disorders [34], and suicide [35], as well as higher rates of PTSD symptomatology [36]. Better positive social support contributes to better mental health status. It can help individuals relieve stress, anxiety, and depressive symptoms [37, 38], and it can improve sleep quality during an outbreak [9, 11]. Thus, better social support can serve as the basis for psychological interventions in the early stage of an epidemic of infectious disease.

Coping styles can affect quality of life of the general population in the face of stress [39, 40]. Among our respondents, negative coping style was associated with higher psychological stress. This is consistent with a previous study that found that individuals were more likely to adopt a negative coping style when exposed to traumatic experiences [41]. Negative coping styles may be related to psychological stress and may contribute to some mental health problems such as anxiety, depression and PTSD [42, 43]. Path analysis also indicates that adopting a negative coping style in response to stressful life events may increase psychological stress [44]. In contrast, a positive coping style means adopting a rational approach to solving a problem, which may promote emotional well-being [45] and protect against mental problems such as depression or suicide [46, 47]. Thus, positive coping style may serve as the basis for psychological interventions in the early stage of an infectious disease epidemic.

There are some advantages in this study. It was conducted timely in the early stages of the COVID-19 epidemic when the fear and uncertainty were widespread and addressed the concern of the general population [48]. The study can help identify the risk factors and provided the basis for the psychological intervention programs, such as promoting social support and positive coping style. This initial evidence from the general Chinese population may be relevant to interventions in other countries for dealing with the COVID-19 and other similar infectious disease outbreak in the future.

There are several limitations in our study. Firstly, the online survey was accessible only to those who could use the Internet. Nevertheless, the WeChat app is widely used in China. Secondly, a potential selection bias existed in our online survey, and snowball sampling also has some demerits (66.91% of our respondents were female) which may reduce the generalizability of the findings to the general Chinese population. Thirdly, we did not assess whether and how respondents were engaging in prevention. Finally, this was a cross-sectional study, so we were unable to follow their mental health status over the course of the COVID-19 epidemic. Therefore, the survey of the requirement of psychological interventions and the long-term psychological implications of infectious disease outbreaks should not be ignored in the future studies.

## Conclusion

Our study suggests that in the general Chinese population, individuals who were younger, female or unmarried, spent more time on the disease, felt greater concern over it, reported lower social support, or showed negative coping style were more likely to suffer higher psychological stress in the early stages of the COVID-19 epidemic. Psychological interventions may target such individuals, who may benefit from better social support and positive coping style in order to face the stresses in the early stages of an infectious disease epidemic.

## Supporting information

S1 Survey The perceived psychological stress and associated factors in the early stages of the coronavirus disease 2019 (COVID-19) epidemic questionnaire. (DOCX)

S1 Data (XLS)

S2 Data (XLSX)
