# Supplementary material for: Perceived psychological stress and associated factors in the early stages of the coronavirus disease 2019 (COVID-19) epidemic: Evidence from the general Chinese population
Source: PLoS One. 2020 Dec 4;15(12):e0243605. doi: 10.1371/journal.pone.0243605 (PMC7717525; doi:10.1371/journal.pone.0243605)
Supplement: S1 Survey — (DOCX) [file pone.0243605.s001.docx]

**S1 Survey. The perceived psychological stress and associated factors in the early stages of the coronavirus disease 2019 (COVID-19) epidemic**

# The perceived psychological stress and associated factors in the early stages of the coronavirus disease 2019 (COVID-19) epidemic Questionnaire

**Demographics:**

1. **Gender**

- Male
- Female

1. **Age**

**_____** years

1. **Marriage status**

- Married
- Unmarried

1. **Education level**

- primary school
- middle school
- high school
- technical qualification
- bachelor’ degree
- graduate

**Instructions: The following questions will ask about your information on the epidemic of COVID-19. Please select the answer of your choice.**

1. **Did you have a visit to Wuhan in the past month?**

- No
- Yes

1. **Was an epidemic occurring in your community in the past month?**

- No
- Yes

1. **How much time did you spend about COVID-19 everyday?**

**_____ hour(s)**

1. **Which of the following matches your concern with media reports related to the epidemic?**

- not concerned
- less concerned
- concerned
- more concerned
- extremely concerned

**Perceived Stress Scale-10 Item:**

**The questions in this scale ask you about your feelings and thoughts during the last month. In each case, please indicate with a check how often you felt or thought a certain way.**

|  | never | almost never | sometimes | fairly often | very often |
| --- | --- | --- | --- | --- | --- |
| 1. In the last month, how often have you been upset because of something that happened unexpectedly? |  |  |  |  |  |
| 2. In the last month, how often have you felt that you were unable to control the important things in your life? |  |  |  |  |  |
| 3. In the last month, how often have you felt nervous and "stressed"? |  |  |  |  |  |
| 4. In the last month, how often have you felt confident about your ability to handle your personal problems? |  |  |  |  |  |
| 5. In the last month, how often have you felt that things were going your way? |  |  |  |  |  |
| 6. In the last month, how often have you found that you could not cope with all the things that you had to do? |  |  |  |  |  |
| 7. In the last month, how often have you been able to control irritations in your life? |  |  |  |  |  |
| 8. In the last month, how often have you felt that you were on top of things? |  |  |  |  |  |
| 9. In the last month, how often have you been angered because of things that were outside of your control? |  |  |  |  |  |
| 10. In the last month, how often have you felt difficulties were piling up so high that you could not overcome them? |  |  |  |  |  |

**Simplified Coping Style Questionnaire (SCSQ):**

**The following lists the attitudes and practices that you may take when you are hit by setbacks or encounter difficulties in your life during the epidemic. Please read each item carefully and then select the corresponding option in each question that matches your situation.**

|  | No use | Occasional use | sometimes use | frequent use |
| --- | --- | --- | --- | --- |
| 1. Relieved through work study or other activities |  |  |  |  |
| 1. Talk with others, express troubles |  |  |  |  |
| 1. Try to see the good side of things |  |  |  |  |
| 1. Change your mind and rediscover what is important in life |  |  |  |  |
| 1. Don't look the problem too seriously |  |  |  |  |
| 1. Stick to your own position and fight for what you want |  |  |  |  |
| 1. Identify different kinds of methods to solve problems |  |  |  |  |
| 1. Ask for advice from relatives, friends or classmates |  |  |  |  |
| 1. Change some of the original practices or some of your own problems |  |  |  |  |
| 1. Learn from others on handling similar difficult situations |  |  |  |  |
| 1. Cultivate hobbies and actively participate in cultural and sports activities |  |  |  |  |
| 1. Try to restrain your disappointment, regret, sadness and anger |  |  |  |  |
| 1. Attempt to take a break or vacation, and temporarily abandon the problem (trouble) |  |  |  |  |
| 1. Relieve trouble by smoking, drinking, taking medicine and eating |  |  |  |  |
| 1. Believe that time will change the status, the only thing to do is to wait |  |  |  |  |
| 1. Trying to forget the whole thing |  |  |  |  |
| 1. Rely on others to solve problems |  |  |  |  |
| 1. Accept reality because there is no other way |  |  |  |  |
| 1. Imagine that one kind of miracle might happen and change the status |  |  |  |  |
| 1. Comfort yourself |  |  |  |  |

**Social support rating scale (SSRS) 社会支持评定量表**

| **Items** |  |  |  |  |
| --- | --- | --- | --- | --- |
| **1. 您有多少关系密切，可以得到支持和帮助的朋友?(只选一项)**  **How many close friends do you have who can get support and help? (Choose only one)** | **一个也没有**  **none of them** | **1-2** | **3-5** | **6个或6个以上/**  **6 or more** |
| **2. 近一年来，您:(只选一项)**  **Over the past year, you (choose only one)** | **远离家人，且独居一室**  **Live away from family and live alone in one room** | **住处经常变动，多数时间和陌生人住在一起**  **Living quarters change frequently and spend most of the time with strangers** | **和同学、同事或朋友住在一起**  **Live with classmates, colleagues or friends** | **和家人住在一起**  **Live with your family** |
| **3. 您与邻居:(只选一项)**  **You and your neighbors :(choose only one)** | **相互之间从不关心，只是点头之交**  **They never cared about each other, they were just nodding acquaintances** | **遇到困难可能稍微关心**  **You may be slightly concerned about difficulties** | **有些邻居很关心您**  **Most of your neighbors care a lot about you** | **大多数邻居都很关心您**  **Most of your neighbors care a lot about you** |
| **4. 您与同事:(只选一项)**  **You and colleagues :(choose only one)** | **相互之间从不关心，只是点头之交**  **They never cared about each other, they were just nodding acquaintances** | **遇到困难可能稍微关心**  **You may be slightly concerned about difficulties** | **有些同事很关心您**  **Some colleagues are concerned about you** | **大多数同事都很关心您**  **Most of your colleagues care a lot about you** |
| **5. 从家庭成员得到的支持和照顾:(在合适的框内划“√”)(只选一项)**  **Support and care from family members :(check √ in the appropriate box)(select only one)** | **无**  **no** | **极少**  **very few** | **一般**  **general support** | **全力支持**  **very support** |
| **A夫妻（恋人）**  **Husband and wife (lovers)** |  |  |  |  |
| **B父母parents** |  |  |  |  |
| **C儿女children** |  |  |  |  |
| **D兄弟姐妹siblings** |  |  |  |  |
| **E其他成员（如嫂子）**  **Other members (e.g. sister-in-law)** |  |  |  |  |
| **6. 过去，您在遇到急难情况时，曾经得到的经济支持或解决实际问题的帮助的来源有:**  **In the past, you have received financial support or help to solve practical problems in emergencies from the following sources:** | **无任何来源**  **Without any source** | **有下列来源（可选多项）A、配偶。B、其他家人。C、朋友。D、亲戚。E、同事。F、工作单位。G、党团工会等官方或半官方组织。H、宗教、社会团体等非官方组织。I、其他。**  **There are the following sources (optional) :A, spouse. B. Other family members. C. Friends. D. Relatives. E. Colleagues. F. Work Unit. G. Official or semi-official organizations such as party, league and trade unions. H. Non-governmental organizations such as religious and social organizations. I. Others.** | | |
| **7. 过去，在您遇到困难或急难情况时，曾经得到的安慰和关心的来源有:**  **In the past, when you encountered difficulties or emergencies, you have received comfort and care from the following sources:** | **无任何来源**  **Without any source** | **有下列来源（可选多项）A、配偶。B、其他家人。C、朋友。D、亲戚。E、同事。F、工作单位。G、党团工会等官方或半官方组织。H、宗教、社会团体等非官方组织。I、其他。**  **There are the following sources (optional) :A, spouse. B. Other family members. C. Friends. D. Relatives. E. Colleagues. F. Work Unit. G. Official or semi-official organizations such as party, league and trade unions. H. Non-governmental organizations such as religious and social organizations. I. Others.** | | |
| **8. 您遇到烦恼时的倾诉方式:(只选一项)**  **How you talk about your troubles :(choose only one)** | **从不向任何人诉说**  **Never told anyone** | **只向关系极为密切的1-2人诉说**  **Only speak to one or two people who are very close to you** | **如果朋友主动询问时说出来**  **Say if your friend asks** | **主动诉说自己的烦恼，以获得支持和理解**  **Take the initiative to share your problems for support and understanding** |
| **9. 您遇到烦恼时的求助方式:(只选一项)**  **How to ask for help when you are in trouble :(choose only one)** | **只靠自己，不接受别人帮助**  **Rely only on yourself, don't accept help from others** | **很少请求别人帮助**  **Seldom ask for help** | **有时请求别人帮助**  **Sometimes ask for help** | **有困难时经常向家人、亲人、组织求援**  **Often ask for help from family members, relatives and organizations when in trouble** |
| **10. 对于团体(如党团组织、宗教组织、工会、学生会等)组织活动，您:(只选一项)**  **For groups (such as party and caucus organizations, religious organizations, trade unions, student unions, etc.) to organize activities, you :(choose only one)** | **从不参加**  **Never participate** | **偶尔参加**  **Occasionally attending** | **经常参加**  **Often take part in** | **主动参加并积极活动**  **Take the initiative to participate and be active** |

**Thank you for participating in this survey.**
